# Prehospital fluid therapy in patients with suspected infection: a survey of ambulance personnel’s practice

**DOI:** 10.1186/s13049-022-01025-1

**Published:** 2022-05-31

**Authors:** Marie Egebjerg Jensen, Arne Sylvester Jensen, Carsten Meilandt, Kristian Winther Jørgensen, Ulla Væggemose, Allan Bach, Hans Kirkegaard, Marie Kristine Jessen

**Affiliations:** 1 Research Center for Emergency Medicine, Aarhus University Hospital, Palle Juul-Jensens Boulevard 99, J103, 8200 Aarhus N, Denmark; 2 Department of Research and Development, Prehospital Emergency Medical Services, Central Denmark Region, Olof Palmes Allé 34, 8200 Aarhus N, Denmark; 3 Prehospital Emergency Medical Services, Central Denmark Region, Olof Palmes Allé 34, 8200 Aarhus N, Denmark; 4 Department of Clinical Medicine, Aarhus University, Incuba Skejby, Palle Juul-Jensens Boulevard 82, 8200 Aarhus N, Denmark

**Keywords:** Emergency Medical Services, Fluid administration, Suspected infection, Guideline, Sepsis, Survey

## Abstract

**Background:**

Fluid therapy in patients with suspected infection is controversial, and it is not known whether fluid treatment administered in the prehospital setting is beneficial. In the absence of evidence-based guidelines for prehospital fluid therapy for patients with suspected infection, Emergency Medical Services (EMS) personnel are challenged on when and how to initiate such therapy.

This study aimed to assess EMS personnel’s decision-making in prehospital fluid therapy, including triggers for initiating fluid and fluid volumes, as well as the need for education and evidence-based guidelines on prehospital fluid therapy in patients with suspected infection.

**Methods:**

An online survey concerning fluid administration in prehospital patients with suspected infection was distributed to all EMS personnel in the Central Denmark Region, including ambulance clinicians and prehospital critical care anaesthesiologists (PCCA). The survey consisted of sections concerning academic knowledge, statements about fluid administration, triggers to evaluate patient needs for intravenous fluid, and clinical scenarios.

**Results:**

In total, 468/807 (58%) ambulance clinicians and 106/151 (70%) PCCA responded to the survey. Of the respondents, 73% (n = 341) of the ambulance clinicians and 100% (n = 106) of the PCCA felt confident about administering fluids to prehospital patients with infections. However, both groups primarily based their fluid-related decisions on “clinical intuition”. Ambulance clinicians named the most frequently faced challenges in fluid therapy as “Unsure whether the patient needs fluid” and “Unsure about the volume of fluid the patient needs”. The five most frequently used triggers for evaluating fluid needs were blood pressure, history taking, skin turgor, capillary refill time, and shock index, the last of which only applied to ambulance clinicians. In the scenarios, the majority administered 500 ml to a normotensive woman with suspected sepsis and 1000 ml to a woman with suspected sepsis-related hypotension. Moreover, 97% (n = 250) of the ambulance clinicians strongly agreed or agreed that they were interested in more education about fluid therapy in patients with suspected infection.

**Conclusion:**

The majority of ambulance clinicians and PCCA based their fluid administration on “clinical intuition”. They faced challenges deciding on fluid volumes and individual fluid needs. Thus, they were eager to learn more and requested research and evidence-based guidelines.

**Supplementary Information:**

The online version contains supplementary material available at 10.1186/s13049-022-01025-1.

## Introduction

Sepsis is one of the most common presentations to Emergency Departments (ED) globally and is associated with significant morbidity and mortality [1–3]. Most sepsis patients are admitted through the ED and approximately 45–75% of these patients are brought in by ambulance by Emergency Medical Services (EMS) [4–6].

Although the definition of sepsis has continually evolved, the emphasis has remained on assessing patients for early diagnosis and treatment, mainly fluid and antibiotics. EMS personnel, as frontline health workers, are important in the early diagnosis and treatment of sepsis [7, 8]. Approximately 30–64% of sepsis patients receive prehospital intravenous fluid [9, 10], usually between 300 and 500 ml [9]. A previous study found that receiving fluid during EMS transportation was associated with reduced in-hospital mortality in patients with sepsis-associated hypotension [11]. However, there is a paucity of evidence concerning prehospital fluid therapy in patients with suspected infection and sepsis, and there is no consensus about the best treatment [12, 13].

This study aimed to investigate fluid treatment in patients with suspected infection by EMS personnel, namely ambulance clinicians and prehospital critical care anaesthesiologists (PCCA). In particular, the study focuses on triggers to initiate fluid therapy and the fluid volumes administered. Furthermore, we aimed to investigate whether EMS personnel feel confident and have the required skills to handle the fluid therapy of patients with suspected infection, sepsis and suspected infection with hypotension, and whether they want to request better evidence and guidelines.

## Methods

### Setting and study participants

The Danish EMS is a two-tiered ambulance system consisting of ambulances as first-line response and an advanced response from PCCAs in rapid-response vehicles or helicopters [14–16].

Healthcare in Denmark is a tax-supported service. Each of the five regions has its own EMS and a varying number of regional general hospitals capable of treating most common medical and surgical conditions. The Central Denmark Region has 69 ambulances staffed by ambulance clinicians (emergency medical technician (EMT) students, ambulance assistants, EMTs, or paramedics) and ten rapid-response vehicles staffed by a PCCA and an EMT or a paramedic.

We identified all EMS personnel in Central Denmark Region from mailing lists provided by the Prehospital EMS, Central Denmark Region and Falck Denmark A/S. The survey was distributed to 810 ambulance clinicians and 152 PCCAs.

The present guideline for prehospital fluid administration in the Central Denmark Region recommends 1000–2000 ml NaCl 0.9% as a fast infusion for septic shock; however, no recommendations for suspected infection or sepsis without hypotension are given.

### Survey instrument

To examine the fluid treatment administered by ambulance clinicians and PCCAs in patients with suspected infection, an online survey was developed.

The survey instrument included both 5-point Likert-scale questions rated from ‘strongly disagree’ to ‘strongly agree’ and multiple-choice options for different parameters. More attitude-based questions included a “Don’t know” option, to ensure, that respondents would complete the survery in stead of abandoning the survey or answering randomly. The survey consisted of three sections: (1) baseline information, (2) perception of, education and knowledge about fluid administration and (3) scenarios about fluid administration (two hypothetical scenarios of patients brought in by EMS). The survey was in Danish. A translated version is provided in the Additional file.

The online survey was developed and refined through a thorough, stepwise process. First, the survey was developed with a group of paramedics and physicians with special knowledge of and interest in fluid therapy, inspired by a similar in-hospital survey which was modified to reflect prehospital challenges: i.e. about fluid administration to patients with suspected infection as patients are in the prehospital setting in stead of a firm diagnosis of sepsis and to cover only the period of transportation to the hospital. Then, the survey was pretested by six EMS employees who volunteered to do so, followed by a 30–60-min semi-structured interview. The interview first covered the volunteers’ perception of the entire field of prehospital fluid administration in general, followed by going though all questions ensuring, they perceived the questions as intended. The interviews resulted in a more exact and improved survey in terms of understanding.

Baseline information covered age, educational level, years of experience and gender. In the section ‘Perception of, education and knowledge about fluid administration’, the respondents were asked how much they agreed to statements about fluid therapy, as well as whether they were aware of and used the current guideline. Afterwards, they had to indicate if they faced challenges and if yes, then which kinds of challenges they faced from a predefined list, with the option to add further challenges as comments. Finally, they were asked to select the five most commonly used triggers, from a list of 22 predefined triggers, for fluid initiation. We created two almost identical clinical scenarios: A 55-year-old, previously healthy woman (70 kg), complaining of fever and dyspnoea was assessed by the ambulance clinicians. For the last 14 days she had been coughing and spitting. She was slightly confused (Glasgow Coma Scale (GCS) 15), blood pressure 120/75, pulse rate 120, respiration frequency 28, temperature: 39.1 Celsius and saturation 92% (3 Litre nasal oxygen per minute). The expected transportation time was 30 min.

In the second, she was hypotensive with a blood pressure of 88/60 mmHg. For each scenario, respondents were asked (1) how much fluid they would administer to the patient during 30 min of transportation, (2) how fast they would infuse and (3) how they made their decision.

### Data collection and procedure

The survey was distributed through emails with an embedded survey link. Emails were sent on June 9th, 2021, with up to six reminders. The survey was closed on July 19th, 2021. Responses were anonymous. Participation was voluntary, and informed consent was obtained at the beginning of the survey. No incentives were offered. Partly completed surveys were excluded from the analysis. All responses were collected through REDCap, version 10.6.16, hosted at Aarhus University [17].

### Data analyses

All data were presented as numbers and proportions (%) for dichotomous and categorical variables. Data were analysed using Stata Version 16 (StataCorp LP, College Station, TX, USA).

## Results

### Survey response and respondents’ characteristics

Of the 962 individuals contacted, 58% (n = 468) ambulance clinicians and 70% (n = 106) PCCAs completed the survey. The distribution of personnel among ambulance clinician respondents were EMT: 49% (n = 279), ambulance assistants: 11% (n = 61), paramedics: 16% (n = 92) and EMT students: 6% (n = 36). The majority in both groups were male. PCCAs were in general older, but had less prehospital experience than ambulance clinicians. The characteristics of the respondents are shown in Table 1.

**Table 1:** Characteristics of the respondents

	Ambulance clinicians^a^	PCCA
	(n = 468)	(n = 106)
Education, n (%)		
EMT-students	36 (8%)	–
Ambulance assistants	61 (13%)	
EMT	279 (59%)	
Paramedics	92 (20%)	
Age, n (%)		
20–30 years	91 (19%)	0 (0%)
31–40 years	133 (28%)	15 (15%)
41–50 years	119 (25%)	61 (58%)
51–60 years	79 (17%)	15 (14%)
61–70 years	46 (10%)	14 (13%)
> 70 years	0 (0%)	0 (0%)
Gender, n (%)		
Male	426 (91%)	70 (66%)
Female	41 (9%)	35 (33%)
Other	1 (0%)	1 (1%)
Prehospital work experience, n (%)		
0–11 months	27 (6%)	7 (7%)
1–2 years	27 (6%)	8 (8%)
3–4 years	38 (8%)	14 (13%)
5–7 years	45 (10%)	29 (27%)
8–12 years	71 (15%)	23 (22%)
12–20 years	102 (22%)	13 (12%)
+ 20 years	158 (34%)	12 (11%)

*EMT* emergency medical technician, *PCCA* prehospital critical care anaesthesiologist

^a^Ambulance assistants, EMT-students, EMTs, Paramedics

### Perception of, education and knowledge about fluid administration

In total, 100% PCCA (n = 106) and 69% ambulance clinicians (n = 321) ‘strongly agreed’ or ‘agreed’ that ‘they feel confident and have the required skills to handle the fluid therapy of patients with sepsis’. The same pattern was found for septic shock (Table 2). Thirty-nine percent (n = 184) of the ambulance clinicians had not received educational sessions about fluid and electrolyte therapy at work in more than three years, and 28% (n = 132) had never received education about this topic at all, whereas this was the case for 41% (n = 44) and 7% (n = 7) for PCCAs, respectively. Meanwhile, 97% (n = 250) of the ambulance clinicians and 67% (n = 71) PCCAs ‘strongly agreed’ or ‘agreed’ they would be interested in more education about fluid therapy in patients with suspected infection, and 26% (n = 119) of ambulance clinicians ‘strongly agreed’ that a more detailed guideline about prehospital fluid therapy would be helpful in their daily work, which was the case for 1% (n = 1) of PCCAs (Table 2).

**Table 2:** Survey responses: confidence in fluid administration and guidelines

	Ambulance clinicians^a^	PCCA
	(n = 468)	(n = 106)
“ I am confident and have the skills to handle fluid treatment of patients with sepsis”, n (%)		
Strongly agree	56 (12%)	71 (67%)
Agree	285 (61%)	35 (33%)
Neither or	100 (21%)	–
Disagree	26 (6%)	–
Strongly disagree	1 (0%)	–
“ I am confident and have the skills to handle fluid treatment of patients with sepsis shock”, n (%)		
Strongly agree	74 (16%)	72 (68%)
Agree	247 (53%)	34 (32%)
Neither or	108 (23%)	–
Disagree	38 (8%)	–
Strongly disagree	1 (0%)	–
The present [local] guideline about fluid administration helps me in my daily work, n (%)		
Strongly agree	13 (3%)	13 (12%)
Agree	210 (45%)	41 (39%)
Neither or	185 (40%)	3 (3%)
Disagree	45 (10%)	–
Strongly disagree	9 (2%)	49 (46%)
Don’t know	6 (1%)	–
A more detailed guideline about fluid treatment would help me in my daily work, n (%)		
Strongly agree	119 (26%)	1 (1%)
Agree	260 (56%)	20 (19%)
Neither or	65 (14%)	36 (34%)
Disagree	18 (4%)	21(20%)
Strongly disagree	2 (0%	3 (3%)
Don’t know	4 (1%)	25 (24%)

*EMT* emergency medical technician, *PCCA* prehospital critical care anaesthesiologist

^a^Ambulance assistants, EMT-students, EMTs, Paramedics

**Fig. 1:**
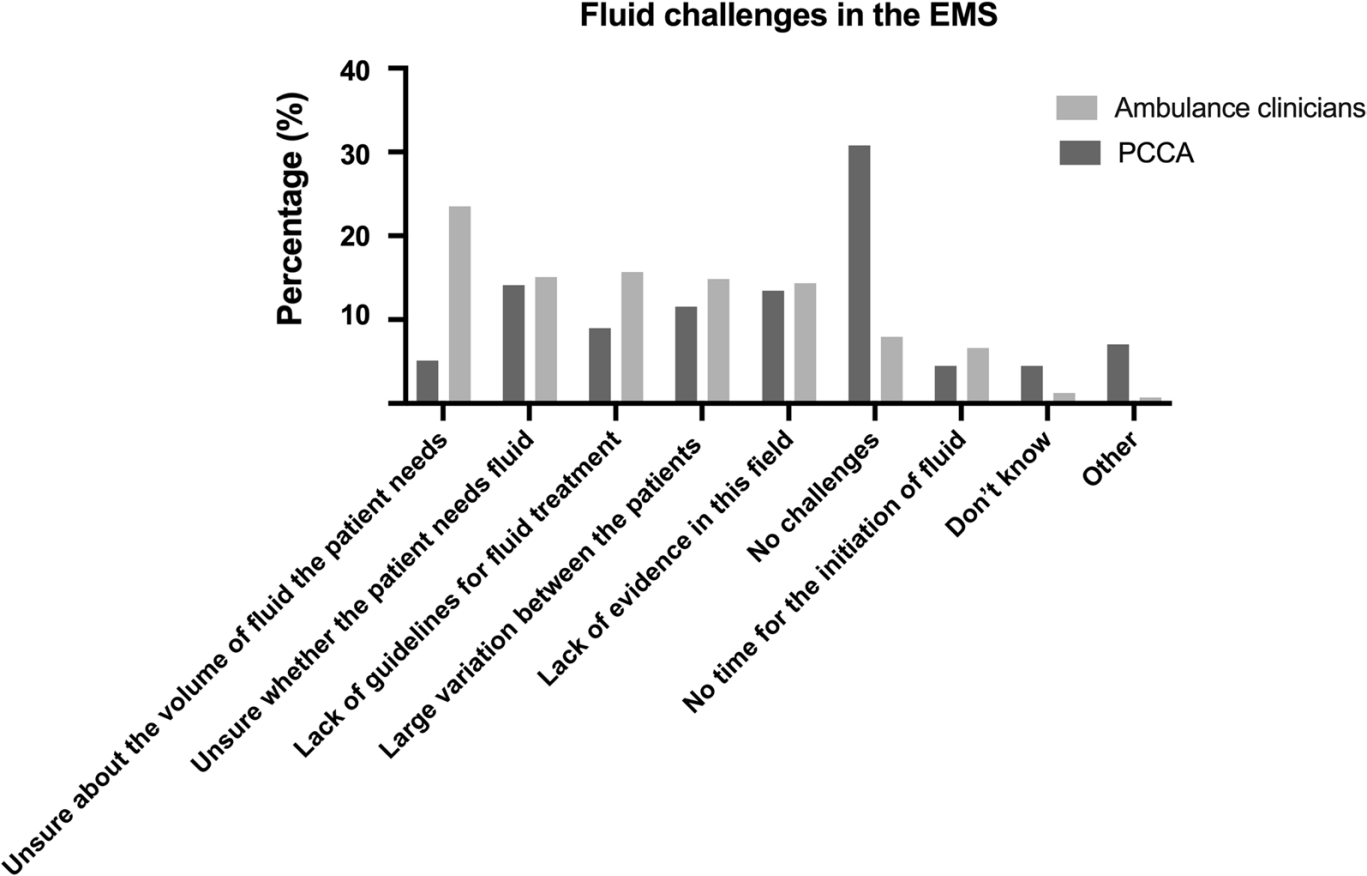
Survey responses: Fluid challenges in the EMS. Bar chart showing fluid challenges in the two respondent groups: Ambulance clinicians and PCCA. EMS: Emergency Medical Services; PCCA: Prehospital critical care anaesthesiologist

The three most commonly faced challenges in fluid therapy for ambulance clinicians were ‘unsure about the volume of fluids the patient needs’, ‘lack of guidelines for fluid therapy’ and ‘unsure whether the patient needs fluid’. The majority of PCCAs stated ‘no challenges’, with 14% (n = 22) being ‘Unsure whether the patient needs fluid’ and 13% (n = 21) citing ‘lack of evidence in this field’ (Fig. 1, Additional file 1: Table S1).

### Statements about fluid administration

The respondents were given six different statements or claims about fluid administration in patients with suspected infection (Additional file 1: Table S1). The majority ‘agreed’ or ‘strongly agreed’ that ‘Intravenous fluid raises the blood pressure in patients with sepsis’ (66% (n = 308) of ambulance clinicians and 71% (n = 75) of PCCAs). The majority of both groups ‘agreed’ or ‘strongly agreed’ with the statement ‘I consider intravenous fluid to be a medication’. Only 5% ambulance clinicians and 1% PCCAs disagreed that ‘intravenous fluid can have side effects’.

Thirty-five percent (n = 166) of the ambulance clinicians, in contrast to 9% (n = 9) in the PCCA group, ‘agreed’ or ‘strongly agreed’ with the statement ‘I have the feeling of at least doing something when I give the patient intravenous fluid—It is better to do something than nothing’. One in four administered intravenous fluids to ensure the placement of the peripheral venous catheter in both groups (Additional file 1: Table S1).

### Triggers to evaluate the patients’ need for intravenous fluid

**Fig. 2:**
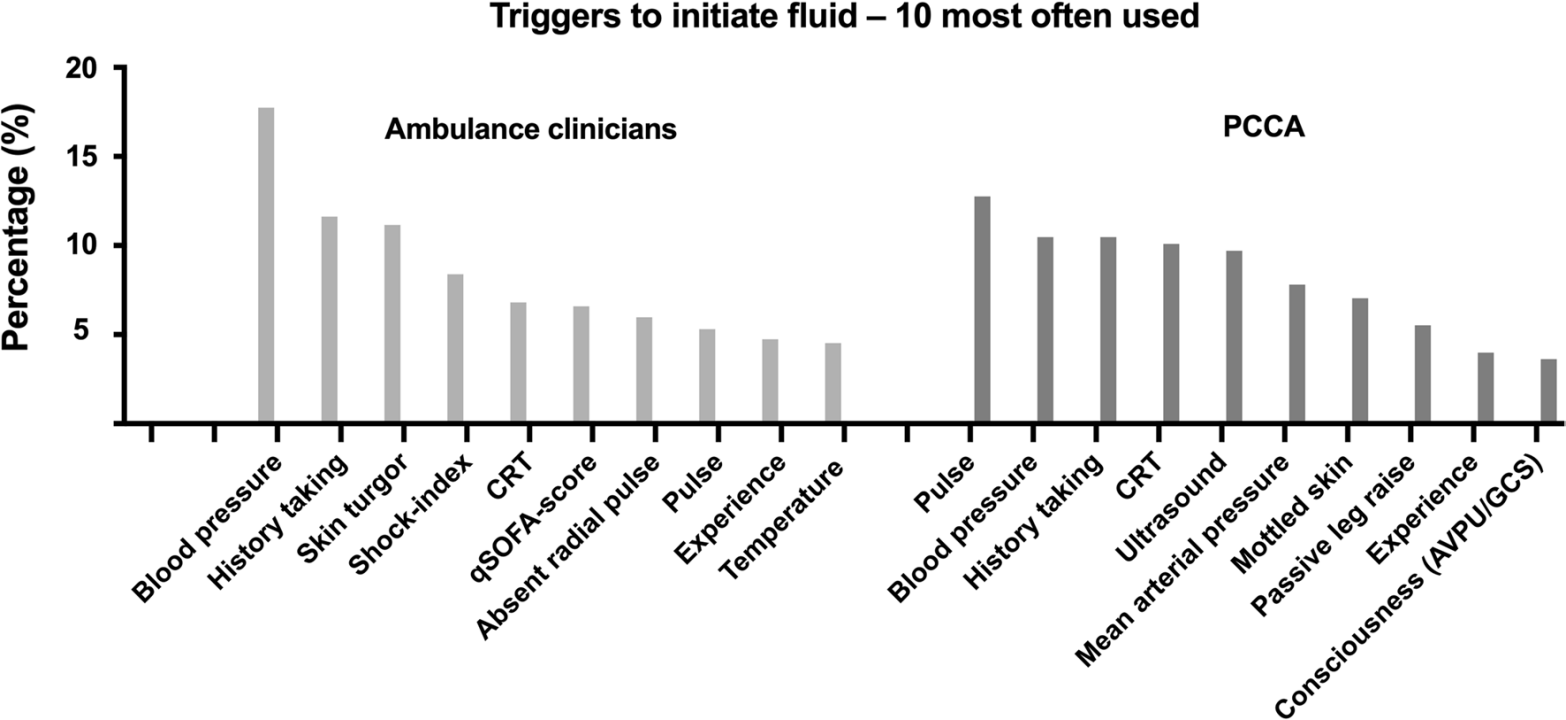
Survey responses: Triggers to initiate fluid–10 most often used. Bar chart showing the ten most used triggers to initiate fluid therapy in sepsis patients in the two respondent groups. Ambulance clinicians: Ambulance assistants, EMTs, Paramedics; PCCA: Prehospital critical care anaesthesiologist; CRT: Capillary Refill Time, qSOFA: quick Sequential Organ Failure Assessment score, AVPU: alert, verbal, pain, unresponsive, GCS: Glasgow Coma Scale

The five most frequently used triggers to assess the patients’ fluid requirement for ambulance clinicians were blood pressure (18% (n = 459)), history taking (12% (n = 320)), skin turgor (11% (n = 271)), shock index (8% (n = 198)) and capillary refill time (7% (n = 208)), while for PCCA they were pulse rate (13% (n = 67)), blood pressure (10% (n = 55)), history taking (10% (n = 55), capillary refill time (10% (n = 55)) and ultrasound of heart, lungs or vena cava inferior (10% (n = 51)). The top ten triggers for ambulance clinicians and PCCA are shown in Fig. 2.

### Fluid administration scenarios

The first clinical scenario described a patient with suspected infection. The majority in both groups chose to administer 500 mL intravenous fluid (ambulance clinicians: 28% (n = 130); PCCA: 40% (n = 41)). The prefered infusion method for ambulance clinicians was slow infusion (> 30 min) (70% (n = 223)); however, fast infusion was often used by PCCAs (n = 35 (45%)).

**Fig. 3:**
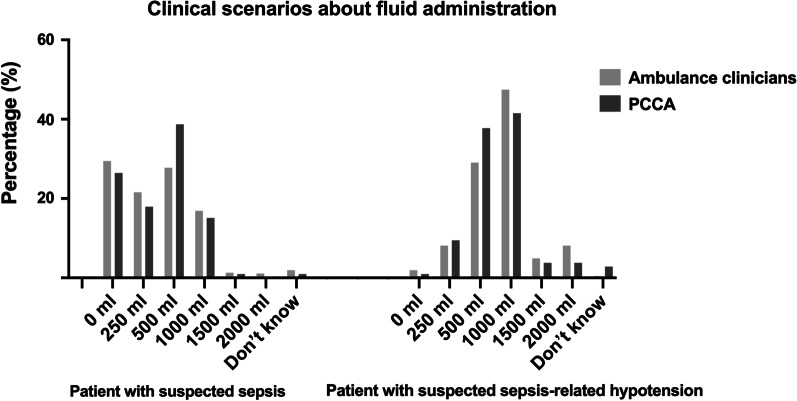
Survey responses: Scenarios about fluid administration. Bar chart showing fluid administration in the two scenarios in the two respondent groups: ambulance clinicians and PCCA. Ambulance clinicians: Ambulance assistants, EMTs, Paramedics; PCCA: Prehospital critical care anaesthesiologist

The patient in second scenario had suspected sepsis-related hypotension. In the second scenario, 47% (n = 222) of the ambulance clinicians and 42% (n = 44) of the PCCA administered 1000 mL intravenous fluid. Ambulance clinicians’ most frequently used infusion method was ‘fast infusion over 15–30 min’ (53% (n = 241)) in this scenario; however, the PCCAs more often chose ‘as fast as possible’ (n = 53 (52%)) (Additional file 1: Table S2). For both scenarios, the decision was based on ‘clinical intuition’ (ambulance clinicians: 49% (n = 230); PCCA: 62% (n = 64)); however, ‘knowledge and evidence’ (13% in case one and 19% in case two) in the PCCA group, and ‘history taking’ in the ambulance clinician group (19% in case one and 12% in case two), were also often used (Fig. 3, Additional file 1: Table S2).

## Research about fluid therapy

Sixty-seven percent (n = 267) of the ambulance clinicians and 51% (n = 55) of PCCAs ‘strongly agreed’ or ‘agreed’ that there is a lack of research into and evidence about prehospital fluid therapy, and 97% vs. 67%, respectively, ‘strongly agreed’ or ‘agreed’ that they were interested in more education about fluid therapy in patients with suspected infection (Table 3).

**Table 3:** Survey responses: education and research

	Ambulance clinicians^a^	PCCA
	(n = 468)	(n = 106)
When was last time you had educational sessions about fluid and electrolyte treatment at your work?		
0–11 months	35 (8%)	16 (15%)
1–2 years	77 (17%)	23 (22%)
+ 3 years	184 (39%)	44 (41%)
Never	132 (28%)	7 (7%)
Don’t know	40 (9%)	17 (16%)
There is a lack of research and evidence in prehospital fluid treatment in patients with suspected infections		
Strongly agree	60 (13%)	9 (8%)
Agree	207 (44%)	46 (43%)
Neither or	118 (25%)	29 (27%)
Disagree	14 (3%)	4 (4%)
Strongly disagree	2 (0%)	1 (1%)
Don’t know	67 (14%)	17 (16%)
I would be interested in more education about fluid administration in patients with infections		
Strongly agree	15 (57%)	15 (14%)
Agree	235 (40%)	56 (53%)
Neither or	42 (3%)	22 (21%)
Disagree	–	7 (7%)
Strongly disagree	–	3 (3%)
Don’t know	–	3 (3%)

*EMT* emergency medical technician, *PCCA* prehospital critical care anaesthesiologist

^a^Ambulance assistants, EMTs, Paramedics

## Discussion

Our survey found that the majority of both ambulance clinicians and PCCAs felt confident about administering fluids to prehospital patients with suspected infection; however, both groups faced challenges in deciding on fluid volumes and individual fluid needs, basing their decisions on clinical intuition. Seventy-two percent of ambulance clinicians and 88% of PCCAs considered fluids to be medication; however, 35% of the ambulance clinicians felt they administered fluids to ‘just do something’ and one in four administered fluids to ensure placement of the peripheral venous catheter. Blood pressure, pulse rate and history taking were the overall most common triggers for administering fluids. Most frequently, 500 mL were administered to a normotensive patient with suspected sepsis and 1000 mL to a hypotensive sepsis patient within 30 min. of transportation, with a more rapid infusion for hypotension. Overall, 67% of the ambulance clinicians strongly agreed or agreed that there is a lack of research and evidence within the field of prehospital fluid therapy of patients with infections.

We found that a higher proportion of respondents felt confident in administering fluid to septic shock than to sepsis patients. This may be due to the fact that the Surviving Sepsis Campaign gives recommendations for fluid administration in sepsis-related hypotension (30 ml/kg) but not for sepsis [18]. Furthermore, the guideline is targeted at in-hospital care and is only a ‘weak recommendation (with) low quality of evidence’ [19, 20]. Therefore, it was surprising that 100% of PCCAs in our study actually felt confident in administering fluids to sepsis patients. Sepsis is a broad definition covering a heterogeneous patient population with a variety of infectious sites. A recent study found that assessment was difficult when signs and symptoms were vague and not confirmed by the guidelines [21]. The heterogeneity and difficult assessment complicates fluid administration to patients with normotensive sepsis. Accordingly, in the current study, both respondent groups regarded the lack of guidelines as a challenge, and ambulance clinicians in particular expressed a need for a more detailed prehospital guideline.

### Triggers of fluid administration

In this study, blood pressure was the most frequently used trigger for initiating fluid therapy in septic patients across both respondent groups and > 60% of respondents in both groups agreed that intravenous fluids raises blood pressure in patients with sepsis. In line with these findings, a study showed that systolic blood pressure ≤ 100 mmHg was strongly associated with the diagnosis of sepsis [22]. Blood pressure in terms of mean arterial pressure (MAP) is also used in the diagnosis of sepsis (using the SOFA score) and septic shock. However, there is no valid evidence that using blood pressure or any other trigger to determine fluid initiation in sepsis patients improves patient outcomes. A study investigating permissive hypotension and vasopressor-use in elderly hypotensive intensive care unit patients found no difference in outcome between the permissive hypotensive group (MAP 60-65 mmHg) and the usual care group [23]. Our study showed that PCCAs mostly used pulse rate, blood pressure and ultrasound of heart, lungs and vena cava inferior, whereas the ambulance clinicians ranked blood pressure first, then history taking and shock index. Some of the differences are probably due to accessibility, as ultrasound is not within the ambulance clinicians’ scope of practice. Furthermore, some triggers were inter-dependent: for example, shock index is pulse divided by systolic blood pressure with a possibility of choosing one of these randomly over the other. In general, the use of different triggers in the respondent groups represents variation in clinical practice, education or culture between ambulance clinicians and PCCAs, with the latter working both prehospital and in-hospital. It would be of interest to conduct more research about which triggers to use for initiating fluid therapy and also as targets to evaluate the response of fluid administration in both hospital and prehospital settings.

Our study found that a larger proportion of ambulance clinicians than PCCAs disagreed that intravenous fluid was medication (13% vs 6%). In line with this finding, 5% of ambulance clinicians disagreed that intravenous fluids could have side effects vs only 1% of PCCAs. Although the numbers in general are small, the differences are noteworthy. The ambulance clinicians are the first EMS personnel to handle and treat the patients; however, follow-up on their patients is not possible after patient handover in the ED. Some side effects from fluid therapy often present later in the course of hospital treatment; thus, the PCCAs, who also work in-hospital, are more prone to experience the side effects than the ambulance clinicians.

### More education

Several studies point towards an insufficient level of knowledge and awareness regarding sepsis care in the EMS [24, 25]. In this study, we did not test respondents’ knowledge of sepsis definitions but asked about previous educational sessions on fluids and sepsis. More than 24% of all respondents had never received education about fluid and electrolyte therapy, and 98% of the ambulance clinicians expressed interest in more education about fluid administration to patients with infections. Overall, ambulance clinicians are eager to learn more, and education focusing on patients with infections and sepsis is wanted. However, given the lack of evidence in this area, it is difficult to conduct proper training and give best recommendations about the treatment of sepsis.

The findings of this survey provide important insights and opportunities to improve the quality of care of patients with suspected infection in the prehospital setting. These findings also provide further support for the development of an educational program for ambulance clinicians, in particular, with an in-depth focus on sepsis and fluid therapy, as requested by the respondents, however again stressing the problem with wanting evidence-based guidelines and education and at the same time lacking substantial scientific evidence—pre- as well as in-hospital.

### Limitations

This study has several strengths and limitations. Our survey was developed to reflect prehospital challenges and piloted for face validity before distribution. The research instrument was validated internally in the prehospital setting in the Central Denmark Region; however, we expect that results could be both used and reproduced in other settings. We rigorously identified all EMS personnel, used reminder emails to improve the survey response rate and achieved an acceptable 63% response rate. These steps suggest the internal validity of our data is high and representative of the Central Denmark Region and Danish practice in the prehospital setting. However, the respondents may represent the fraction of prehospital personnel who are interested in this field and topic and, thus, introduce bias. Furthermore, the hypothetical scenarios were limited by the clinical detail that could be included and the responses may not represent actual daily practice i.e. it is not from this survey possible to determine what actually happens in the prehospital setting in terms of fluid administration to patients with suspected infections.

## Conclusion

The majority of ambulance clinicians and PCCAs based their fluid administration on 'clinical intuition'. They faced challenges in deciding on fluid volumes and individual fluid needs. In general, respondents were eager to learn more and requested research and evidence-based guidelines within the field.

## Supplementary Information

**Additional file 1**. Prehospital fluid therapy in patients with suspected infection: a survey of ambulance.

## Data Availability

The de-identifyed data can be shared, please contact marie.jessen@rm.dk.

## Abbreviations

ED: Emergency department
EMS: Emergency medical services
EMT: Emergency medical technicians
PCCA: Prehospital critical care anaesthesiologists
