# Supplementary material for: Prehospital fluid therapy in patients with suspected infection: a survey of ambulance personnel’s practice
Source: Scand J Trauma Resusc Emerg Med. 2022 May 31;30:38. doi: 10.1186/s13049-022-01025-1 (PMC9158174; doi:10.1186/s13049-022-01025-1)
Supplement: Supplementary file 1 — Additional file 1. Prehospital fluid therapy in patients with suspected infection: a survey of ambulance. [file 13049_2022_1025_MOESM1_ESM.docx]

**Additional file 1 for:**

**“Prehospital fluid therapy in patients with suspected infection: a survey of ambulance**

**personnel’s practice”**

**Questionnaire**

Title: Prehospital fluid administration in patients with suspected infection

1. **Introduction:**

How old are you?

- <20 years
- 20-30 years
- 31-40 years
- 41-50 years
- 51-60 years
- 61-70 years
- >70 years

What is your gender?

- Male
- Female
- Other

I am working as

- Emergency Medical Technician (EMT)-student
- Ambulance Assistant
- EMT
- Paramedic
- Prehospital critical care anesthesiologist (PCCA)

My primary employment is at:

- Præhospitalet Region Midt (Prehospital Services Central Denmark Region)
- Response
- Falck
- Samsø Redningskorps

How many years of prehospital experience do you have?

- 0-11 months
- 1-2 years
- 3-4 years
- 5-7 years
- 8-12 years
- 12-20 years
- +20 years

**2) Academic knowledge about fluid administration**

*Statements about fluid administration*

I have a feeling of at least doing something when administering intravenous fluid. It is better to do something than nothing.

Strongly agree

Agree

Neither or

Disagree

Strongly disagree

Intravenous fluid raises the blood pressure in patients with sepsis

Strongly agree

Agree

Neither or

Disagree

Strongly disagree

The treatment for sepsis includes intravenous fluid

Strongly agree

Agree

Neither or

Disagree

Strongly disagree

I consider intravenous fluid to be a medication

Strongly agree

Agree

Neither or

Disagree

Strongly disagree

Intravenous fluid can have side effects

Strongly agree

Agree

Neither or

Disagree

Strongly disagree

I administer intravenous fluid to ensure that the placed peripheral venous catheter “stays safe”

Strongly agree

Agree

Neither or

Disagree

Strongly disagree

*Statements about your skills*

I am confident and have the skills to handle fluid treatment of patients with sepsis

Strongly agree

Agree

Neither or

Disagree

Strongly disagree

Don’t know

I am confident and have the skills to handle fluid treatment of patients with sepsis shock

Strongly agree

Agree

Neither or

Disagree

Strongly disagree

Don’t know

Comments?

*Definition of sepsis: suspected infection+ organ dysfunction## Definition of sepsis shock: suspected infection+ lactat >2mmol+the need for Noradrenalin or similar*

Do you experience challenges in fluid treatment in the prehospital setting?

- No challenges
- Unsure whether the patient needs fluid
- No time for the initiation of fluid
- Lack of guidelines for fluid treatment
- Lack of evidence in this field
- Heterogeneity among patients with suspected infection
- Unsure about the volume of fluid the patient needs
- Don’t know
- Other

*Guidelines*

- The current guideline about fluid administration is useful in my daily work.

Strongly agree

Agree

Neither or

Disagree

Strongly disagree

Don’t know

- The current guideline is based on evidence

Strongly agree

Agree

Neither or

Disagree

Strongly disagree

Don’t know

- A more detailed guideline about fluid treatment would be useful in my daily work.

Strongly agree

Agree

Neither or

Disagree

Strongly disagree

Don’t know

*Daily fluid administration*

Choose the 5 parameters that you most you most frequently use triggers to assess the patients’ fluid requirement

- Blood pressure
- History taking
- Skin turgor
- Capillary Refill Time
- Shock-index
- Pulse
- qSOFA-score
- Absent radial pulse
- Experience
- Mean arterial pressure
- Temperature (measured rectal, oral or ear(tympanic))
- SIRS-criteria
- Respiration frequency
- Consciousness (AVPU/GCS)
- Ultrasound of heart, lungs, or vena cava inferior
- Edemas
- Mottled skin
- Passive leg raise
- Mucous membrane
- Stethoscopia pulmonalis
- Temperature of the extremities
- Jugular vein distension

Do you use other parameters than these to decide how much fluid the patient should be given? …

- **Scenarios about fluid administration**

*Scenario 1*

A 55-years-old, previous healthy woman (70kg), who was picked up by ambulance clinicians because of fever and dyspnea. The last 14 days she had been coughing and spitting. She is slightly confused (GCS 15), blood pressure 120/75, pulse 120, respiration frequency 28, temperature: 39,1 Celsius and saturation 92% (3 Liter nasal oxygen per minute). The expected transportation time is 30 min.

How much fluid would you give this patient before reaching the hospital?

- No fluid
- 250 ml
- 500 ml
- 1000 ml
- 1500 ml
- 2000 ml
- Don’t know.

Which infusion method would you use?

- As fast as possible
- Fast infusion over 15-30 min.
- Slow infusion, +30min
- Don’t know

What did you use in your decision making?

- Knowledge and evidence
- Clinical intuition
- Education
- Experience
- History taking
- Instructions
- Other

Please elaborate?

*Scenario 2*

A 55-years-old, previous healthy woman (70kg), who was picked up by ambulance clinicians because of fever and dyspnea. The last 14 days she had been coughing and spitting. She is slightly confused (GCS 15), blood pressure 88/60, pulse 120, respiration frequency 28, temperature: 39,1 Celsius and saturation 92% (3 Liter nasal oxygen per minute). The expected transportation time is 30 min.

How much fluid would you give this patient before reaching the hospital?

- No fluid
- 250 ml
- 500 ml
- 1000 ml
- 1500 ml
- 2000 ml
- Don’t know.

Which infusion method would you use?

- As fast as possibly
- Fast infusion over 15-30 min.
- Slow infusion, +30min
- Don’t know

What did you use in your decision making?

- Knowledge and evidence
- Clinical intuition
- Education
- Experience
- History taking
- Instructions
- Other

Please elaborate?

- **Education and research**

When was the last time you received scheduled lessons/instructions/education about fluid and electrolyte treatment at your work?

- 0-11 months
- 1-2 years
- >3 years
- Never
- Don’t know

There is a lack of research and evidence in prehospital fluid treatment in patients with suspected infection.

Strongly agree

Agree

Neither or

Disagree

Strongly disagree

Don’t know

I would be interested in more education about fluid administration and resuscitation of patients with infection.

Strongly agree

Agree

Neither or

Disagree

Strongly disagree

Don’t know

- **Comments**

| **Table S1** Statements and claims about fluid administration | | |  |
| --- | --- | --- | --- |
|  | **Ambulance clinicians***  **(N=468)** | **PCCA**  **(N=106)** | |
| “I have the feeling of at least doing something when I give the patient intravenous fluid. It is better to do something than nothing.”, n (%)  Strongly agree  Agree  Neither or  Disagree  Strongly disagree | 27 (5%)  139 (30%)  136 (29%)  112 (24%)  54 (12%) | 1 (1%)  8 (8%)  26 (25%)  32 (30%)  39 (37%) | |
| “Intravenous fluid raises the blood pressure in patients with sepsis.”, n (%)  Strongly agree  Agree  Neither or  Disagree  Strongly disagree | 43 (9%)  265 (57%)  117 (25%)  38 (8%)  4 (1%) | 9 (9%)  66 (62%)  31 (29%)  -  - | |
| “The treatment for sepsis includes intravenous fluid”, n (%)  Strongly agree  Agree  Neither or  Disagree  Strongly disagree | 173 (37%)  263 (56%)  24 (5%)  5 (1%)  2 (0%) | 44 (42%)  56 (53%)  6 (6%)  -  - | |
| “I consider intravenous fluid to be a medication”, n (%)  Strongly agree  Agree  Neither or  Disagree  Strongly disagree | 138 (30%)  197 (42%)  69 (15%)  50 (11%)  14 (3%) | 55 (52%)  38 (36%)  7 (7%)  4 (4%)  2 (2%) | |
| “Intravenous fluid can have side effect”, n (%)  Strongly agree  Agree  Neither or  Disagree  Strongly disagree | 193 (41%)  229 (49%)  24 (5%)  26 (4%)  6 (1%) | 76 (72%)  25 (24%)  4 (4%)  1 (1%)  - | |
| “I administer intravenous fluid to ensure that the placed peripheral venous catheter “stays safe””, n (%)  Strongly agree  Agree  Neither or  Disagree  Strongly disagree | 42 (9%)  72 (16%)  119 (25%)  143 (31%)  92 (20%) | 6 (6%)  17 (16%)  21 (20%)  38 (36%)  24 (23%) | |
| *****Assistants, EMTs, Paramedics | | | |

| **Table S2: Clinical scenarios** | | |
| --- | --- | --- |
| Scenario 1: A 55-years-old, previous healthy woman (70kg), who was picked up by ambulance clinicians because of fever and dyspnea. The last 14 days she had been coughing and spitting. She is slightly confused (GCS 15), BP 120/75, PR 120, RR 28, temperature: 39,1 Celsius and saturation 92% (3 Liter nasal oxygen per minute). The expected transportation time is 30 min. | | |
|  | **Ambulance clinicians***  **(N=468)** | **PCCA**  **(N=106)** |
| How much fluid would you give this patient before you reach the hospital?  0 ml  250 ml  500 ml  1000 ml  1500 ml  2000 ml  Don’t know | 138 (29%)  101 (22%)  130 (28%)  79 (17%)  6 (1%)  5 (1%)  9 (2%) | 28 (27%)  19 (18%)  41 (40%)  16 (15%)  1 (1%)  0 (0%)  1 (1%) |
| What kind of infusion method would you use?  As fast as possible  Fast infusion in 15-30 min.  Slow infusion in >30min  Don’t know | n=319  7 (2%)  86 (27%)  223 (70%)  3 (1%) | n=77  13 (17%)  35 (45%)  29 (38%)  0 (0%) |
| What did you use as a basis in this decision making?  Knowledge and evidence  Clinical intuition  Education  Experience  History taking  Guidelines  Other | n=459  15 (3%)  235 (51%)  42 (9%)  34 (7%)  85 (19%)  36 (8%)  12 (3%) | n=105  13 (13%)  69 (66%)  0 (0%)  7 (7%)  12 (12%)  0 (0%)  4 (4%) |
| Scenario 2: Blood pressure was 88/66 otherwise identical case | | |
| How much fluid will you give this patient before you reach the hospital?  0 ml  250 ml  500 ml  1000 ml  1500 ml  2000 ml  Don’t know | n=466  9 (2%)  38 (8%)  136 (29%)  222 (47%)  23 (5%)  38 (8%)  2 (0%) | n=103  1 (1%)  10 (9%)  40 (38%)  44 (42%)  4 (4%)  4 (4%)  3 (3%) |
| What kind of infusion method would you use?  As fast as possible  Fast infusion in 15-30 min.  Slow infusion in >30min  Don’t know | n=455  106 (23%)  241 (53%)  105 (23%)  3 (1%) | n=102  53 (52%)  46 (45%)  2 (2%)  1 (1%) |
| What did you use as a basis in this decision making?  Knowledge and evidence  Clinical intuition  Education  Experience  History taking  Guidelines  Other | n=466  24 (5%)  230 (49%)  55 (12%)  34 (7%)  54 (12%)  61 (13%)  8 (2%) | n=103  19 (19%)  64 (62%)  0 (0%)  7 (7%)  7 (7%)  1 (1%)  5 (5%) |
| *****Assistants, EMTs, Paramedics. GCS: Glasgow Coma Score, BP: blood pressure, PR: Pulse rate, RF: respiratory rate | | |
